# The relationship between influenza vaccine hesitancy and vaccine literacy among youth and adults in China

**DOI:** 10.3389/fimmu.2024.1444393

**Published:** 2024-08-05

**Authors:** Li Wang, Mengjie Guo, Yan Wang, Ren Chen, Xiaolin Wei

**Affiliations:** ^1^ School of Health Service Management, Anhui Medical University, Hefei, Anhui, China; ^2^ Dalla Lana School of Public Health, University of Toronto, Toronto, ON, Canada; ^3^ Institute of Health Policy, Management, and Evaluation, Dalla Lana School of Public Health, University of Toronto, Toronto, ON, Canada

**Keywords:** vaccine hesitancy, vaccine literacy, influenza vaccine, vaccination, residents

## Abstract

**Objectives:**

The present study aimed to assess influenza vaccine hesitancy and vaccine literacy levels among youth and adults in China, as well as the association between them.

**Methods:**

An online cross-sectional survey was conducted in Mainland China. Participants’ total vaccine literacy and three sub-dimension vaccine literacy (knowledge literacy, competence literacy, and decision-making literacy) were assessed by a validated vaccine literacy scale. Having received influenza vaccination in the past three years or intending to accept it in next influenza season indicates less influenza vaccine hesitancy.

**Results:**

Among 997 participants, a sub-optimal vaccine literacy was observed, with a mean score of 66.83 ± 10.27. Regression models 1–4 revealed that participants with middle (*aOR:* 1.431, *P*=0.039, *95% CI:* 1.018~2.010) or high (*aOR:* 1.651, *P*=0.006, *95% CI:* 1.157~2.354) total vaccine literacy, as well as those with high competence literacy (*aOR:* 1.533, P=0.017, *95% CI:* 1.079~2.180), or high decision-making literacy (*aOR:* 1.822, *P*=0.001, *95% CI:* 1.261~2.632) were more likely to have been vaccinated against influenza at least once in past three years. However, those with a high knowledge literacy were associated with a lower influenza vaccine rate (*aOR:* 0.676, *P*=0.046, *95% CI:* 0.460~0.994). Regression models 5–8 revealed that participants with middle (*aOR:* 1.661, *P*=0.008, *95% CI:* 1.142~2.414) or high total vaccine literacy (*aOR:* 2.645, *P*=0.000, *95% CI:* 1.774~3.942), as well as those with middle (*aOR:* 1.703, P=0.005, *95% CI:* 1.177~2.464) or high competence literacy (*aOR:* 2.346, *P*=0.000, *95% CI:* 1.159~3.461), or high decision-making literacy (*aOR:* 2.294, *P*=0.000, *95% CI:* 1.531~3.436) were more likely to express the willingness to receive the influenza vaccine in the next influenza season.

**Conclusion:**

The participants’ influenza vaccine hesitancy was negatively associated with their total vaccine literacy levels and two of the three sub-dimensions: competence literacy and decision-making literacy. Knowledge literacy suggested a positive or no relationship with influenza vaccine hesitancy.

## 1 Introduction

Playing a crucial role in protecting public health and preventing the spread of infectious diseases, vaccination stands out as a key primary prevention measure (1). However, vaccine hesitancy is giving rise to refusal or delayed vaccination in the presence of vaccine availability. Even those who have been vaccinated as required may still harbor doubts, leading to fluctuations in vaccination coverage (2). This hesitation could potentially impact the implementation of preventive vaccination, and it has been listed as one of the top ten global health threats by the World Health Organization (WHO) (3). The definition of vaccine literacy drew inspiration from the term “health literacy”. Ratzan SC (4), in 2011, initially proposed the notion of vaccine literacy, which emphasized individuals’ capacity to acquire, comprehend, and utilize fundamental vaccination information and services, while also evaluating the potential consequences and risks of their actions to make informed health choices. Vaccine literacy, managing to transfer effective information and facilitate necessary dialog, has been seen as a promising strategy in tackling vaccine hesitancy (5–7).

However, vaccine hesitancy determinants often overlook the significance of inadequate vaccine literacy. This constraint further undermines the effectiveness of vaccination communication efforts (2, 8). Although vaccine literacy is not the only influencing factor in vaccination acceptance, enhancing people’s vaccine literacy and diminishing their vaccine hesitancy remains crucial in promoting vaccination.

Influenza is an acute respiratory infectious infection caused by seasonal influenza virus (9). Influenza caused a large number of cases and deaths worldwide through its seasonal wave of infection (10). The Global Influenza Surveillance and Response System (GISRS) reported that even in mild years, influenza can result in 290,000 to 650,000 fatalities, 3 million to 5 million severe cases worldwide, and create more social and economic damage when it is an epidemic (11, 12). The influenza vaccine is a key tool in combating seasonal influenza and its related risks (9). When the circulating virus aligned well with the vaccine, influenza vaccinations were believed to lower the risk of contracting the illness by 40% to 60% (13). However, influenza vaccine hesitancy posed a significant obstacle to worldwide endeavors aimed at alleviating the impact of both seasonal and pandemic influenza (14). The global influenza vaccination rate among the general public was low, with many countries, including the USA, Canada, Europe, and Australia, reporting that influenza vaccine coverage in target populations below the 75% recommended by the World Health Organization (15–19). Additionally, influenza vaccination rates were much lower in middle-low income countries/regions compared to high-income countries/regions (20). Influenza vaccination rates among Chinese residents were notably low. It was found that influenza vaccination rates for general population, individuals with chronic diseases, healthcare workers were 16.74%, 14.12%, and 23.07%, respectively (20). Currently, the influenza vaccine has not been incorporated into China’s National Expanded Programme on Immunization (NEPI), rendering it a self-funded and optional vaccination in the country. According to the Technical Guidelines for Influenza Vaccination in China (2022~2023), it was recommended that individuals aged 6 months and older, willing to receive influenza vaccine and without contraindications, should be vaccinated against influenza (21). However, the implementation of these recommendations faced significant challenges due to influenza vaccine hesitancy (22–24). Certain major cities, such as Beijing and Shenzhen, have enacted policies offering free influenza vaccines to specific high-risk groups, including senior citizens, healthcare workers, and children. However, influenza vaccination rates in these places were still low. For example, influenza vaccination rates among frail old people in Beijing urban communities were just about 20%, and only 4.8% of 5045 elderly with chronic diseases in Shenzhen were immunized against influenza (25, 26). It may suggest that attention should be paid not only to improving affordability but also to enhancing people’s vaccine literacy to alleviate vaccine hesitancy.

The effect of vaccine literacy on public vaccine confidence in China has aroused researchers’ attention (27, 28). The relationship between vaccine literacy and vaccination behavior was systematically reviewed, indicating the significance of exploring a more appropriate research tool for evaluating vaccine literacy (5). Most of the research tools served to measure vaccine literacy were intended for general health literacy, rather than being developed specifically for assessing vaccine literacy. The frequently used vaccine literacy scale, Literacy about Vaccination of Adults in Italian (HLVa-IT), which involves 14 items in total and encompasses both functional and interactive-critical dimensions, was developed from the Ishikawa, originally applied to measure health literacy among patients with chronic diseases (29, 30). Vaccine Health Literacy Scale and COVID-19-VLS, containing similar constructs, could be seen as some adaptations of Ishikawa (31, 32). Yang (33) applied the China-HLVa-IT questionnaire to explore its association with vaccine hesitancy in China. It was also an adaptation of HLVa-IT, sharing similar psychometric constructs, including functional, interactive/communicative, and critical items, with a focus on information capacity (33).

The ability to process information is a crucial factor influencing individual’s vaccination decision. However, a number of potential determinants were identified as being associated with vaccine coverage, including factors based on rational information processing such as carefully calculating or comparing the risks or benefits of vaccines, as well as factors related to simple heuristics that allow people to make decisions quickly and intuitively (34, 35). That could be one of the main reasons why no conclusive evidence demonstrating a consistent association between health literacy or vaccine literacy and vaccine acceptance. The association has been shown to be positive, while according to others, it is negative or non-existent (31, 36–42). It may suggest that more specific assessment methods should be focused to better understand the causal relationship between vaccine literacy and vaccine hesitancy (5, 43). Hou (44) and Meng (45) built indicator systems for evaluating vaccine confidence and vaccine literacy in China, including dimensions such as basic knowledge, fundamental beliefs, and behavioral capabilities, providing valuable inspiration and a foundation for understanding Chinese public’s vaccine literacy, even though the items were general and abstract. Wang (46) constructed a vaccine literacy scale tailored for community residents in China (China-VLS), not only including the core components of existing tools such as the ability to acquire, understand, and apply information, but also integrating other crucial determinants such as vaccine health beliefs, and vaccination related decision-making literacy. The focus of this tool is on factors influencing vaccine hesitancy from the perspective of individual behavior decision-making, allowing for a deeper observation of the relationship between vaccine literacy and vaccine hesitancy. The aim of the current study was to assess vaccine literacy among Chinese community residents using China-VLS, and to explore the relationship between participants’ vaccine literacy and their influenza vaccine hesitancy.

## 2 Materials and methods

### 2.1 Study design and participants

A self-developed survey questionnaire was utilized on the Credamo JianShu platform to conduct a survey. Credamo is a professional data collection company with 3,000,000 members spanning every province and administrative region in Mainland China. Its panel members were sourced from diverse channels, encompassing offline customers and residents, college campuses (including students and faculty), businesses (including employers and users), as well as previous participants of its offline surveys (47). Credamo distributed survey links to 1000 randomly selected panel members aged over 18 years old for each month from July to September, 2023. Data collection stopped after reaching a predetermined about 1000 participants who completed the survey. A total of 997 observations were retained after manually removing 3 participants who failed 1 attention-checking questions created by the researchers. Effective questionnaire recovery rate was 97%.

### 2.2 Study tool

The questionnaire encompassed demographic information (address, age, education level, marital status, occupation, family income, chronic illness status), questions related to influenza vaccine hesitancy, as well as a validated vaccine literacy scale for Chinese community residents, China-VLS. The construction of China-VLS was discussed in another paper published by the same research team (46). The validated vaccine literacy scale applied in the present study paid more attentions to the key factors influencing vaccine hesitancy, including but not limited to the indicators in other vaccine literacy evaluation tool. Information abilities were the main focus in most research tools used to evaluate vaccine literacy in other studies. The philosophy behind this is the rational decision-making model. However, a number of potential determinants were identified as being associated with vaccine acceptance, including factors based on rational information processing such as carefully calculating or comparing the risks or benefits of vaccines, as well as factors related to simple heuristics that allow people to make decisions quickly and intuitively (34, 35). Sometimes, the latter may play a major role in deciding whether to vaccinate oneself or a child (35). That could be one of main reasons why no conclusive evidence demonstrating a consistent association between health literacy or vaccine literacy skills and vaccine acceptance.

The process of identifying the indicators for the vaccine literacy scale was guided, but not limited to, three main kinds of theoretical frameworks (1): Health Behavior Theories such as Knowlege-Attitude-Practice Theory (KAP) and The Health Belief Model (HBM), which have been utilized in past vaccination behavior research (48). KAP focuses on the impact of objective knowledge on health practices through attitude change, while HBM focuses on the impact of subjective health beliefs on health behaviors. They have often been employed to uncover misconceptions or misunderstandings that could impede the implementation of desired activities and serve as potential barriers to health behavior change (49, 50). (2): “3C” Model, proposed by the SAGE of the WHO to analyze the influencing factors of vaccine hesitancy. “3C” refers to three dimensions: Confidence, Convenience and Complacency (51). (3): The existing framework of vaccine literacy scales used in other studies. Most of the research tools served to measure vaccine literacy were intended for general health literacy, rather than being developed specifically for assessing vaccine literacy. And also they were always designed with the perspective of the competence to find, understand and judge information related to vaccine and vaccination.

A total of 30 items were developed through a two-round Delphi expert panel consensus. The three first-level indicators were “knowledge literacy”, “competence literacy”, and “decision-making literacy”. “Knowledge literacy” corresponded to the knowledge dimension in the KAP model, covering items related to general and objective knowledge about vaccines and vaccinations, including vaccine features, vaccination procedures, and policy regulations based on the *Vaccine Management Law in China*. “Competence literacy” centered around information literacy, which served as the core of most existing vaccine literacy scales. It aimed to assess participants’ information processing ability around vaccines and vaccination. Additionally, action capability and payment capability were also involved in “Competence literacy”. “Decision-making literacy” mainly involved health beliefs about vaccines in general, and partly referencing the “3C” model. Four second-level of indicators, disease prevention self-satisfaction, trust, compliance, and collective responsibility were involved in “Decision-making literacy”.

The Cronbach’s alpha for this scale was 0.658, which was greater than 0.6, indicating good internal consistency. The Bartlett’s test of sphericity yielded a value of 502.796 (*P* < 0.001), and the Kaiser-Meyer-Olkin (KMO) test value was 0.764, also greater than 0.6. Three common factors were extracted (eigenvalues all > 1), with a cumulative variance contribution rate of 73.568%.

For each item, a score of one point was initially awarded if the participant’s response aligned with the predetermined correct or reasonable options; otherwise, no points were awarded. Subsequently, conversion was performed using the difficulty coefficient method. The difficulty coefficient was calculated as the reciprocal of the correct response rate for each item, with a lower correct response rate corresponding to a higher difficulty coefficient and, consequently, a higher score for correctly answering that item. Finally, the total scale score for each participant was transformed into a 100-point system. The scores of the three sub-dimension vaccine literacy scales were calculated as well (46).

Based on the participants’ scale scores from high to low, we divided the total vaccine literacy scores and the scores of each sub-dimension of the vaccine literacy into three levels: low, medium, and high, with roughly equal numbers of people in each group. This categorization facilitated group comparisons and logistic regression analysis.

There were two questions about influenza vaccine hesitancy: one was about past influenza vaccination behavior (Have you ever been vaccinated against influenza in the past three years)?, and the other was future influenza vaccination willingness (Will you receive influenza vaccine in the next influenza season)?. Having received influenza vaccination in the past three years or intending to accept it in next influenza season indicated less influenza vaccine hesitancy.

### 2.3 Statistical analysis

Stata SE17 was used for statistical analysis. Descriptive statistics were used to analyze participants’ general characteristics and their vaccine literacy level.

Bivariate analysis included one independent variable and one dependent variable for each group comparison. Categorical variables were presented as counts and percentages (%), and group comparisons were conducted using the chi-square test. Participants’ influenza vaccination rate and the percentage of participants willing to receive the influenza vaccine were the dependent variables, respectively. The total vaccine literacy level, each sub-dimension vaccine literacy level, each socio-demographic variable, and each perception regarding influenza and the influenza vaccine were regarded as independent variables. Each independent variable was analyzed separately with participants’ influenza vaccination rate (see **Table 1**). Similarly, each independent variable was analyzed separately with the percentage of participants willing to receive the influenza vaccine (see **Table 2**).

**Table 1:** Univariate analysis of participants’ influenza vaccination behavior over the past three years and their vaccine literacy levels, socio-demographic variables, as well as perceptions regarding influenza and influenza vaccine.

Category	No. (%) of subjects	No. Vaccinated	Coverage rate ( %)	*x^2^*	*P*
**Total Vaccine Literacy Level**				8.706	0.013*
Low	332(33.30)	183	55.12		
Middle	330(33.10)	207	62.73		
High	335(33.60)	221	65.97		
**Knowledge Literacy Level**				8.334	0.016*
Low	284(28.49)	184	64.79		
Middle	477(47.84)	301	63.10		
High	236(23.67)	126	53.39		
**Competence Literacy Level**				15.452	0.000*
Low	331(33.20)	175	52.87		
Middle	350(35.11)	224	64.00		
High	316(31.69)	212	67.09		
**Decision-making Literacy Level**				13.107	0.001*
Low	344(34.50)	198	57.56		
Middle	330(33.10)	189	57.27		
High	323(32.40)	224	69.35		
**Residence**				0.875	0.350
Rural	197(19.76)	115	58.38		
Urban	800(80.24)	496	62.00		
**Gender**				9.778	0.002*
Female	758(76.03)	444	58.58		
Male	239(23.97)	167	69.87		
**Age group**				0.233	0.629
18-24	642(64.39)	397	61.84		
25 and above	355(35.61)	214	60.28		
**Education**				6.806	0.009*
Bachelor and above	893(89.57)	535	59.91		
Below Bachelor	104(10.43)	76	73.08		
**Marital Status**				1.311	0.252
Married	156(15.65)	102	65.38		
Unmarried/Other	841(84.35)	509	60.52		
**Area**				17.122	0.000*
East	538(55.87)	299	55.58		
Middle	201(29.39)	142	70.65		
West	258(15.55)	170	65.89		
**Occupation**				3.944	0.139
Students	549(55.87)	322	58.65		
Corporate Employees or others	293(29.39)	186	63.48		
Government and Public Institution Staff	155(15.55)	103	66.45		
**Family Monthly Income**				10.596	0.014*
<5000	185(18.56)	96	51.89		
5000-10000	436(43.73)	285	65.37		
10001-20000	264(26.48)	158	59.85		
>20000	122(11.23)	72	64.29		
**How concerned are you about getting the flu?**				23.976	0.000*
No Concern	173(17.35)	80	46.24		
Partially concern	419(42.03)	256	61.10		
Concern	405(40.62)	275	67.90		
**Based on your physical condition, if you are infected with the flu, do you think the situation will be serious?**				8.238	0.016*
Not severe	142(14.24)	73	51.41		
Partially severe	666(66.80)	412	61.86		
Severe	189(18.96)	126	66.67		
**Are you worried about the side effects of getting the flu shot?**				1.608	0.448
Worried	359(36.01)	221	61.56		
Partially worried	505(50.65)	315	62.38		
Not worried	133(13.34)	75	56.39		
**Do you agree that the flu vaccine is effective in preventing the flu?**				31.625	0.000*
Not agree	179(17.95)	83	46.37		
Partially agree	596(59.78)	364	61.07		
Agree	222(22.27)	164	73.87		

**Table 2:** Univariate analysis of influenza vaccination willingness in the next influenza season and their vaccine literacy levels, socio-demographic variables, as well as perceptions regarding influenza and influenza vaccine.

Category	No. (%) of subjects	No. Having influenza vaccination willingness	rate( %)	*x^2^*	*P*
**Total Vaccine Literacy Level**				37.013	0.000*
Low	332(33.30)	129	38.86		
Middle	330(33.10)	170	51.52		
High	335(33.60)	209	62.39		
**Knowledge Literacy Level**				6.792	0.034*
Low	284(28.49)	154	54.23		
Middle	477(47.84)	251	52.62		
High	236(23.67)	103	43.64		
**Competence Literacy Level**				48.965	0.000*
Low	331(33.20)	119	35.95		
Middle	350(35.11)	191	54.57		
High	316(31.70)	198	62.66		
**Decision-making Literacy Level**				50.836	0.000*
Low	344(34.50)	153	44.48		
Middle	330(33.10)	138	41.82		
High	323(32.40)	217	67.18		
**Residence**				0.732	0.392
Rural	197(19.76)	95	48.22		
Urban	800(80.24)	413	51.63		
**Gender**				5.103	0.024*
Female	758(76.03)	371	48.94		
Male	239(23.97)	137	57.32		
**Age group**				1.792	0.181
25 and above	642(64.39)	317	49.38		
18-24	355(35.61)	191	53.80		
**Education**				9.677	0.002*
Bachelor and above	893(89.57)	440	49.27		
Below Bachelor	104(10.43)	68	65.38		
**Marital Status**				5.553	0.018*
Unmarried/Other	841(84.35)	415	49.35		
Married	156(15.65)	93	59.62		
**Area**				14.172	0.001*
East	538(55.87)	246	45.72		
Middle	201(29.39)	121	60.20		
West	258(15.55)	141	54.65		
**Occupation**				7.831	0.020*
Students	549(55.87)	260	47.36		
Corporate Employees or others	293(29.39)	156	53.24		
Government and Public Institution Staff	155(15.55)	92	59.35		
**Family Monthly Income**				2.928	0.403
<5000	185(18.56)	87	47.03		
5000-10000	436(43.73)	230	52.75		
10001-20000	264(26.48)	139	52.65		
>20000	122(11.23)	52	46.43		
**How concerned are you about getting the flu?**				35.558	0.000*
No concern	173(17.35)	58	33.53		
Partially concern	419(42.03)	206	49.16		
Concern	485(40.62)	244	60.25		
**Based on your physical condition, if you are infected with the flu, do you think the situation will be serious?**				5.685	0.058
Not severe	142(14.24)	60	42.25		
Partially severe	666(66.80)	344	51.65		
Severe	189(18.96)	104	55.03		
**Are you worried about the side effects of getting the flu shot?**				5.050	0.080
Worried	359(36.01)	167	46.52		
Partially worried	505(50.65)	274	54.26		
Not worried	133(13.34)	67	50.38		
**Do you agree that the flu vaccine is effective in preventing the flu?**				96.575	0.000*
Not agree	179(17.95)	44	24.58		
Partially agree	596(59.78)	300	50.34		
Agree	222(22.27)	164	73.87		

Multivariate analysis included a single dependent variable along with multiple independent variables, covariates, or controlling variables. We applied four multivariate logistic regression models (Models 1–4) to explore the association between influenza vaccination rate and the total vaccine literacy level, as well as each sub-dimension vaccine literacy level, respectively. The analyses were adjusted for socio-demographic variables, as well as perceptions regarding influenza and the influenza vaccine. Only statistically significant variables were presented in **Table 3**. Specifically, Model 1 assessed the association between influenza vaccination rate and the total vaccine literacy levels. Models 2–4 assessed the association between influenza vaccination rate and each sub-dimension vaccine literacy levels, respectively.

**Table 3:** Multivariate logistic regression analyses of participants’ influenza vaccination behavior over the past three years and their vaccine literacy levels, socio-demographic variables, as well as perceptions regarding influenza and influenza vaccine (N = 997).

Category	Model 1		Model 2		Model 3		Model 4	
	*OR (95% CI)*	*P*	*OR (95% CI)*	*P*	*OR (95% CI)*	*P*	*OR (95% CI)*	*P*
Total VL Level								
Low	1.000		NA	NA	NA	NA	NA	NA
Middle	1.431(1.018~2.010)	0.039*	NA	NA	NA	NA	NA	NA
High	1.651(1.157~2.354)	0.006*	NA	NA	NA	NA	NA	NA
Knowledge VL Level								
Low	NA	NA	1.000		NA	NA	NA	NA
Middle	NA	NA	0.922(0.663~1.282)	0.629	NA	NA	NA	NA
High	NA	NA	0.676(0.460~0.994)	0.046*	NA	NA	NA	NA
Competence VL Level								
Low	NA	NA	NA	NA	1.000		NA	NA
Middle	NA	NA	NA	NA	1.392(0.995~1.946)	0.053	NA	NA
High	NA	NA	NA	NA	1.533(1.079~2.180)	0.017*	NA	NA
Decision-making VL Level								
Low	NA	NA	NA	NA	NA	NA	1.000	
Middle	NA	NA	NA	NA	NA	NA	1.094(0.778~1.539)	0.606
High	NA	NA	NA	NA	NA	NA	1.822(1.261~2.632)	0.001*
Gender								
Female	1.000		1.000		1.000		1.000	
Male	1.606(1.143~2.256)	0.006*	1.574(1.120~2.212)	0.009*	1.556(1.107~2.188)	0.011*	1.592(1.132~2.238)	0.007*
Age group								
25 and above	1.000		1.000		1.000		1.000	
18-24	1.692(1.128~2.539)	0.011*	1.676(1.118~2.511)	0.012*	1.664(1.110~2.495)	0.014*	1.672(1.114~2.510)	0.013*
Education								
Bachelor and above	1.000		1.000		1.000		1.000	
Below Bachelor	1.970(1.190~3.259)	0.008*	1.875(1.131~3.108)	0.015*	1.954(1.182~3.233)	0.009*	2.013(1.217~3.330)	0.006*
Area								
East	1.000		1.000		1.000		1.000	
Middle	2.181(1.496~3.179)	0.000*	2.112(1.452~3.073)	0.000*	2.128(1.462~3.096)	0.000*	2.150(1.475~3.134)	0.000*
West	1.659(1.187~2.320)	0.003*	1.591(1.138~2.224)	0.007*	1.661(1.187~2.323)	0.003*	1.626(1.163~2.274)	0.004*
Occupation								
Students	1.000		1.000		1.000		1.000	
Corporate Employees or others	1.636(1.096~2.442)	0.016*	1.636(1.096~2.442)	0.016*	1.650(1.105~2.462)	0.014*	1.596(1.068~2.385)	0.023*
Government and Public Institution Staff	1.779(1.107~2.860)	0.017*	1.770(1.103~2.840)	0.018*	1.768(1.101~2.840)	0.018*	1.769(1.098~2.849)	0.019*
Family Monthly Income								
<5000	1.000		1.000		1.000		1.000	
5000-10000	1.815(1.237~2.662)	0.002*	1.939(1.323~2.840)	0.001*	1.850(1.262~2.711)	0.002*	1.913(1.303~2.807)	0.001*
10001-20000	1.518(0.988~2.323)	0.057*	1.680(1.096~2.576)	0.017*	1.552(1.012~2.381)	0.044*	1.560(1.042~2.455)	0.032*
>20000	2.094(1.220~3.596)	0.007*	2.362(1.377~4.054)	0.002*	2.137(1.247~3.663)	0.006*	2.314(1.348~3.970)	0.002*
How concerned are you about getting the flu?								
No concern	1.000		1.000		1.000		1.000	
Partially concern	1.954(1.294~2.951)	0.001*	1.909(1.263~2.886)	0.002*	1.908(1.262~2.885)	0.002*	1.960(1.295~2.966)	0.001*
Concern	2.523(1.608~3.958)	0.000*	2.430(1.548~3.812)	0.000*	2.409(1.535~3.783)	0.000*	2.489(1.582~3.917)	0.000*
Do you agree that the flu vaccine is effective in preventing the flu?								
Not agree	1.000		1.000		1.000		1.000	
Partially agree	1.687(1.177~2.420)	0.004*	1.793(1.253~2.567)	0.001*	1.710(1.193~2.450)	0.003*	1.700(1.184~2.441)	0.004*
Agree	3.016(1.921~4.735)	0.000*	3.185(2.036~4.983)	0.000*	2.956(1.879~4.650)	0.000*	2.857(1.816~4.493)	0.000*

*①Model 1 assessed the association between influenza vaccination behavior over the past three years and the total vaccine literacy levels, adjusting for socio-demographic variables as well as perceptions regarding influenza and the influenza vaccine. Model 2-4 assessed the association between influenza vaccination behavior over the past three years and the three sub-dimension vaccine literacy levels, respectively, adjusting for socio-demographic variables as well as perceptions regarding influenza and the influenza vaccine.

②NA meant “not applicable” because the relevant variable was not involved in the corresponding regression model.

Similarly, we applied another four multivariate logistic regression models (Models 5–8) to explore the association between the percentage of participants willing to receive influenza vaccine and the total vaccine literacy level, as well as each sub-dimension vaccine literacy level, respectively. The analyses were adjusted for socio-demographic variables, as well as perceptions regarding influenza and the influenza vaccine. Only statistically significant variables were presented in **Table 4**. Specifically, Model 5 assessed the association between the percentage of participants willing to receive the influenza vaccine in next influenza season and the total vaccine literacy levels. Models 6–8 assessed the association between the percentage of participants willing to receive the influenza vaccine in next influenza season and each sub-dimension vaccine literacy level, respectively.

**Table 4:** Multivariate logistic regression analyses of influenza vaccination willingness in the next influenza season and their vaccine literacy levels, socio-demographic variables, as well as perceptions regarding influenza and influenza vaccine (N = 997).

Category	Model 5		Model 6		Model 7		Model 8	
	*OR (95% CI)*	*P*	*OR (95% CI)*	*P*	*OR (95% CI)*	*P*	*OR (95% CI)*	*P*
Total VL Level								
Low	1.000		NA	NA	NA	NA	NA	NA
Middle	1.661(1.142~2.414)	0.008*	NA	NA	NA	NA	NA	NA
High	2.645(1.774~3.942)	0.000*	NA	NA	NA	NA	NA	NA
Knowledge VL Level								
Low	NA	NA	1.000		NA	NA	NA	NA
Middle	NA	NA	0.955(0.668~1.364)	0.800	NA	NA	NA	NA
High	NA	NA	0.8196(0.533~1.256)	0.360	NA	NA	NA	NA
Competence VL Level								
Low	NA	NA	NA	NA	1.000		NA	NA
Middle	NA	NA	NA	NA	1.703(1.177~2.464)	0.005*	NA	NA
High	NA	NA	NA	NA	2.346(1.590~3.461)	0.000*	NA	NA
Decision-making VL Level								
Low	NA	NA	NA	NA	NA	NA	1.000	
Middle	NA	NA	NA	NA	NA	NA	0.893(0.611~1.306)	0.561
High	NA	NA	NA	NA	NA	NA	2.294(1.531~3.436)	0.000*
Area								
East	1.000		1.000		1.000		1.000	
Middle	1.650(1.104~2.467)	0.015*	1.582(1.068~2.346)	0.022*	1.623(1.090~2.417)	0.017*	1.632(1.094~2.435)	0.016*
West	1.367(0.944~1.978)	0.098	1.300(0.902~1.875)	0.160	1.369(1.945~1.982)	0.097	1.309(0.904~1.896)	0.154
How concerned are you about getting the flu?								
No concern	1.000		1.000		1.000		1.000	
Partially concern	2.206(1.354~3.594)	0.001*	2.123(1.308~3.447)	0.002*	2.113(1.296~3.443)	0.003*	2.237(1.364~3.669)	0.001*
Concern	3.759(2.222~6.358)	0.000*	3.482(2.067~5.865)	0.000*	3.427(2.025~5.797)	0.000*	3.588(2.112~6.095)	0.000*
Are you worried about the side effects of getting the flu shot?								
Worried	1.000		1.000		1.000		1.000	
Partially worried	1.434(1.016~2.025)	0.040*	1.704(1.216~2.388)	0.002*	1.592(1.133~2.237)	0.007*	1.552(1.096~2.196)	0.013*
**Not worried**	1.101(0.644~1.882)	0.724	1.459(0.870~2.448)	0.152	1.329(0.785~2.251)	0.290	1.103(0.639~1.905)	0.724
Do you agree that the flu vaccine is effective in preventing the flu?								
Not agree	1.000		1.000		1.000		1.000	
Partially agree	2.764(1.794~4.256)	0.000*	3.057(1.988~4.670)	0.000*	2.783(1.807~4.286)	0.000*	2.951(1.911~4.557)	0.000*
Agree	7.068(4.199~11.896)	0.000*	7.859(4.684~13.184)	0.000*	6.851(4.050~11.590)	0.000*	6.903(4.088~11.659)	0.000*
Have you ever been vaccinated against influenza in the past three years?								
No	1.000		1.000		1.000		1.000	
Yes	6.062(4.386~8.379)	0.000*	6.121(4.450~8.420)	0.000*	6.005(4.355~8.280)	0.000*	5.959(4.088~8.241)	0.000*

*①Model 5 assessed the association between influenza vaccination willingness in next influenza season and the total vaccine literacy levels, adjusting for socio-demographic variables as well as perceptions regarding influenza and the influenza vaccine. Model 6-8 assessed the association between influenza vaccination willingness in next influenza season and the three sub-dimension vaccine literacy levels, respectively, adjusting for socio-demographic variables as well as vaccination behavior in the past three years, and the perceptions regarding influenza and the influenza vaccine.

②NA meant “not applicable” because variable was not involved in corresponding regression model.

Odds ratios (ORs) with 95% confidence intervals (CIs) were calculated using logistic regression analysis for categorical variables. For all analyses, a significance threshold of p-value < 0.05, indicating a two-tailed test, was employed.

## 3 Results

### 3.1 General information about participants

Among the 997 participants, the majority resided in urban areas (800 cases, 80.24%). The participants were predominantly female (758 cases, 76.03%). Since the age group of 18–39 years was the most represented, consisting of 960 individuals (96.29% of the sample), it may be inappropriate to categorize multiple age groups, as seen in other studies. The age of 24 was regarded as an age boundary between youth and adulthood by United Nations (52). The study divided participants into two age groups: those aged 24 years or younger and those older than 24 years. A significant portion had attained a bachelor’s degree or higher (893 cases, 89.57%)^1^, were unmarried (841 cases, 84.35%), and belonged to three types of occupations: including personnel from government or public institutions (155 cases, 15.55%), employees of corporate companies or others (293 cases, 29.39%), and students (549 cases, 55.07%). The majority of participants fell within the household income range of 5000–10000 (436 cases, 43.73%) or 10001–20000 (264 cases, 26.48%). We categorized the participants’ geographical locations into three groups: East (538 cases, 53.96%), Middle (201 cases, 20.16%), and West (258 cases, 25.88%), based on the locations of their provinces in China. These geographical differences could indicate distinct climates that were consistently associated with the influenza epidemic (53). 611 out of 977 participants (61.28%) have received at least one shot of influenza vaccine over the past three years, and 508 (50.95%) participants express the willingness to receive influenza vaccine in the next influenza season (**Table 5**).

**Table 5:** General information about participants.

Category	No. (%) of subjects
**Number of participants**	977
**Age (mean ± SD)**	24.87 ± 6.26
Age group	
18-24	642(64.39)
25 and above	355(35.61)
Residence	
Rural	197(19.76)
Urban	800(80.24)
Gender	
Female	758(76.03)
Male	239(23.97)
Education	
Bachelor and above	893(89.57)
Below Bachelor	104(10.43)
Marital Status	
Married	156(15.65)
Unmarried/Other	841(84.35)
Area	
East	538(53.96)
Middle	201(20.16)
West	258(25.88)
Occupation	
Students	549(55.07)
Corporate Employees or others	293(29.38)
Government and Public Institution Staff	155(15.55)
Family Monthly Income	
<5000	185(18.56)
5000-10000	436(43.73)
10001-20000	264(26.48)
>20000	122(11.23)
Influenza Vaccine Hesitancy	
Have you ever been vaccinated against influenza in the past three years?	
Yes	611(61.28)
No	386(38.72)
Will you receive influenza vaccine in the next influenza season?	
Yes	489(49.05)
No	508(50.95)

### 3.2 Participants’ vaccine literacy

The mean vaccine literacy score of the 997 participants was 66.83 ± 10.27. The mean scores of the three sub-dimension vaccine literacy scales – knowledge literacy scale included 15 items, competence literacy scale included 8 items, and decision-making literacy scale included 7 items – were 33.42 ± 5.17(score range: 20.74~43.18), 17.82 ± 4.61(score range: 2.53~24.70), and 15.48 ± 6.68(score range: 0.00~31.99), respectively.

### 3.3 Bivariate analysis

#### 3.3.1 The correlation between influenza vaccination and vaccine literacy levels, socio-demographic variables, and perceptions regarding influenza and the influenza vaccine

Univariate analysis showed a significant correlation between the respondents’ acceptance of the influenza vaccine over the past three years and the total vaccine literacy levels, as well as the three sub-dimensional vaccine literacy levels (*P*<0.05). A higher influenza vaccination rate was significantly associated with high or middle total vaccine literacy (*x^2^ = * 8.706, *P*<0.05) and high or middle levels of two of the three sub-dimensional vaccine literacy: competence literacy (*x^2^* = 15.452, *P*<0.001) and decision-making literacy (*x^2^ = * 13.107, *P*<0.05). However, a lower influenza vaccination rate was significantly associated with high knowledge literacy (*x^2^ = * 8.334, *P*<0.05) (see **Table 1**).

There was a significant relationship between influenza vaccination rate and certain socio-demographic variables, such as gender (*x^2^* = 9.778, *P*<0.05), education level (*x^2^* = 6.806, *P*<0.05), residing area (*x^2^* = 17.122, *P*<0.001), and family monthly income (*x^2^* = 10.596, *P*<0.05) (see **Table 1**).

A significant relationship was also found between the influenza vaccination rate and some of the participants’ perceptions regarding influenza and the influenza vaccine, such as their level of concern about contracting the flu (*x^2^* = 23.976, *P*<0.001), the perceived severity of being infected with influenza (*x^2^* = 8.238, *P*<0.05), and the perceived effectiveness of the influenza vaccine (*x^2^* = 31.625, *P*<0.05) (see **Table 1**).

#### 3.3.2 The correlation between the willingness to receive the influenza vaccine, vaccine literacy levels, socio-demographic variables, and perceptions regarding influenza and the influenza vaccine

Univariate analysis also indicated a significant correlation between the participants’ willingness to receive the influenza vaccine in the next influenza season and the total vaccine literacy levels, as well as three sub-dimensional vaccine literacy levels (*P*<0.05). A higher willingness to receive the influenza vaccine was significantly associated with high or middle total vaccine literacy (*x^2^* = 37.013, *P*<0.001) and high or middle levels of two of the three sub-dimensional vaccine literacies: competence literacy (*x^2^* = 48.965, *P*<0.001) and decision-making literacy (*x^2^* = 50.836, *P*<0.001). However, a lower willingness to receive the influenza vaccine was significantly associated with high knowledge literacy (*x^2^* = 6.792, *P*<0.05) (see **Table 2**).

There was a significant relationship between the participants’ willingness to receive the influenza vaccine and certain socio-demographic variables, such as gender (*x^2^* = 5.013, *P*<0.05), education level (*x^2^* = 9.677, *P*<0.05), marital status (*x^2^* = 5.553, *P*<0.05), residing area (*x^2^* = 14.172, *P*<0.001). (see **Table 2**)

A significant relationship was also found between the participants’ willingness to receive the influenza vaccine and their perceptions regarding influenza and influenza vaccine, such as their level of concern about contracting the flu (*x^2^* = 35.558, *P*<0.001), and the perceived effectiveness of the influenza vaccine (*x^2^* = 96.575, *P*<0.001) (see **Table 2**).

### 3.4 Multivariate analysis

#### 3.4.1 The multivariate logistic regression analyses of the relationship between influenza vaccination and vaccine literacy levels, socio-demographic variables, and perceptions regarding influenza and the influenza vaccine

The multivariate logistic regression models 1–4 assessed the association between influenza vaccination behavior over the past three years and both the total vaccine literacy levels and the three sub-dimension vaccine literacy levels, respectively. All these analyses adjusted for socio-demographic variables, as well as perceptions regarding influenza and the influenza vaccine. Only statistically significant variables were presented in **Table 3**.

Participants with middle (*aOR:* 1.431, *P*=0.039, *95% CI:* 1.018~2.010) or high (*aOR:* 1.651, *P*=0.006, *95% CI:* 1.157~2.354) total vaccine literacy, or those with high competence literacy (*aOR:* 1.533, *P*=0.017, *95% CI:* 1.079~2.180), or high decision-making literacy (*aOR:* 1.822, *P*=0.001, *95% CI:* 1.261~2.632) were more likely to be vaccinated against influenza at least once in the past three years. However, participants with a high knowledge literacy were associated with a lower influenza vaccination rate (*aOR:* 0.676, *P*=0.046, *95% CI:* 0.460~0.994) (see **Table 3**).

In addition, the multivariate logistic regressions showed a significant relationship between past influenza vaccination behavior and certain variables about socio-demographic variables. For example, Model 1 indicated that males (*aOR:* 1.606, *P*=0.006, *95% CI:* 1.143~2.256), participants aged 25 and above(*aOR:* 1.692, *P*=0.011, *95% CI:* 1.128~2.539), those with an education level below a bachelor’s degree (*aOR:* 1.970, *P*=0.008, *95% CI:* 1.190~3.259), residents of the Middle (*aOR:* 2.181, *P*=0.000, *95% CI:* 1.496~3.179), or West areas (*aOR:* 1.667, *P*=0.003, *95% CI:* 1.192~2.332), corporate employees or others (*aOR:* 1.636, *P*=0.016, *95% CI:* 1.096~2.442), staff of government and public institutions (*aOR:* 1.779, *P*=0.017, *95% CI:* 1.107~2.860), and those with a family monthly income of 5000–10000 (*aOR:* 1.815, *P*=0.002, *95% CI:* 1.237~2.662) or above 20000 (*aOR:* 2.094, *P*=0.007, *95% CI:* 1.220~3.596) had higher times greater likelihood of receiving the influenza vaccine compared to females, participants aged 18–24, those with a bachelor’s degree or above, residents of the East area, students, and those with a family monthly income of less than 5000, respectively. Models 2–4 showed similar results (see **Table 3**).

It was also found in the multivariate logistic regressions a significant relationship between past influenza vaccination behavior and perceptions regarding influenza and the influenza vaccine. Model 1 indicated that participants who partially concerned (*aOR:* 1.954, *P*=0.001, *95% CI:* 1.294~2.951) or concerned (*aOR:* 2.523, *P*=0.000, *95% CI:* 1.608~3.958) about getting influenza, and those who partially agreed (*aOR:* 1.687, *P*=0.004, *95% CI:* 1.177~2.420) or agreed (*aOR:* 3.016, *P*=0.000, *95% CI:* 1.921~4.735) with the effectiveness of the influenza vaccine were more likely to be vaccinated against influenza in the past three years, compared to those who had no concern about getting influenza, and those who disagreed with the effectiveness of the influenza vaccine. Models 2–4 showed similar results (see **Table 3**).

#### 3.4.2 The multivariate logistic regression analyses of the relationship between the willingness to receive the influenza vaccine, vaccine literacy levels, socio-demographic variables, and perceptions regarding influenza and the influenza vaccine

The multivariate logistic regression models 5–8 assessed the association between the willingness to receive the influenza vaccine in the next influenza season and both the total vaccine literacy levels and the three sub-dimension vaccine literacy levels, respectively. All these analyses adjusted for socio-demographic variables, as well as perceptions regarding influenza and the influenza vaccine. Only statistically significant variables were presented in **Table 4**.

Participants who had middle (*aOR:* 1.661, *P*=0.008, *95% CI:* 1.142~2.414) or high total vaccine literacy (*aOR:* 2.645, *P*=0.000, *95% CI:* 1.774~3.942), or middle (*aOR:* 1.703, *P*=0.005, *95% CI:* 1.177~2.464) or high competence literacy (*aOR:* 2.346, *P*=0.000, *95% CI:* 1.159~3.461), or high decision-making literacy (*aOR:* 2.294, *P*=0.000, *95% CI:* 1.531~3.436) were more likely to show willingness to receive influenza vaccine in the next influenza season (see **Table 4**).

There were no significant associations between participants’ willingness to receive the influenza vaccine and most of their socio-demographic variables. However, there was a significant association between influenza vaccination willingness and those who resided in the Middle area (*aOR:* 1.650, *P*=0.015, *95% CI:* 1.104~2.467, in Model 5), compared to those who resided in the East area. Models 6–8 showed similar results (see **Table 4**).

In addition, the multivariate logistic regressions revealed a significant relationship between the willingness to receive the influenza vaccine in the next influenza season and perceptions regarding influenza and the influenza vaccine, along with their vaccination history within the previous three years. Model 5 indicated that participants who partially concerned (*aOR:* 2.206, *P*=0.001, *95% CI:* 1.354~3.594) or concerned (*aOR:* 3.759, *P*=0.000, *95% CI:* 2.222~6.358) about getting influenza, those who partially worried about side effects of influenza vaccine (*aOR:* 1.434, *P*=0.040, *95% CI:* 1.016~1.025), and those who partially agreed (*aOR:* 2.764, *P*=0.000, *95% CI:* 1.794~4.256) or agreed (*aOR:* 7.068, *P*=0.000, *95% CI:* 4.199~11.896) with the effectiveness of the influenza vaccine, and those who had received influenza vaccine in the past three years (*aOR:* 6.062, *P*=0.000, *95% CI:* 4.386~8.379) had higher willingness of receiving influenza vaccine in the next influenza season, compared to those who had no concern about getting influenza, who worried about the side effects of influenza vaccine, who disagreed with the effectiveness of the influenza vaccine, and who had not received influenza vaccine, respectively. Models 6–8 showed similar results (see **Table 4**).

## 4 Discussion

The study indicated that about 60% of the participants had a history of influenza vaccination in the past three years. In recent years, studies showed that the Chinese public’s influenza vaccination rate in a single year was about 20% (54–57). And the influenza vaccination rates in some high-risk population group including health-care workers, children, and senior citizens were about 50% (58–60). Therefore, it was argued that the influenza vaccination rate demonstrated in the study was roughly consistent with that found in other studies. And the influenza vaccination rate was higher than those observed before the COVID-19 pandemic. About a 2% influenza vaccination rate among the Chinese public was reported in 2018 (61, 62). Numerous studies indicated that Chinese residents had a clear shift in attitudes toward influenza vaccination, with more enthusiasm for influenza vaccination, under the background of the COVID-19 epidemic, and COVID-19 vaccination history was associated with the positive actions of influenza vaccination during the pandemic (55, 63). However, the study found that only about 50% of participants expressed willingness to be vaccinated against influenza in the next influenza season. It is essential to pay significant attention to whether the influenza vaccine rate and public enthusiasm, heightened by COVID-19, would decrease as the threat of COVID-19 diminishes and people’s apprehensions subside.

Using a validated vaccine literacy scale, the present study showed a sub-optimal vaccine literacy among participants, with a mean score of 66.83 ± 10.27 (full score was set as 100). A modified HLVa-IT scale was used to assess Chinese residents’ vaccine literacy and demonstrated that the mean vaccine literacy value was 52.16 ± 8.93 (full score was set as 75) among 7731 participants (33). However, the construction and content of these two types of scales differed, although they shared some similar indicators or descriptions. The HLVa-IT scale was developed in the Italian context and has been expanded to other countries, primarily focusing on evaluating participants’ ability to handle vaccine-related information across three dimensions: functional, interactive, and critical vaccine literacy (29). The vaccine literacy scale used in the study was more comprehensive and tailored for Chinese community residents, not only including indicators regarding information ability but also other indicators related to vaccine knowledge literacy and decision-making literacy. And it might be the first to list decision-making literacy as an independent dimension on the vaccine literacy scale and find its positive association with influenza vaccination. Vaccine hesitancy referred to an uncertainty condition about a vaccination decision (64). Vaccine hesitancy was marked by fluctuations in mental states, particularly in emotional responses. In the context of decision-making, it’s essential to distinguish between the affective nature of vaccine hesitancy and its behavioral manifestations (33). Vaccination behavior factors, including information ability, vaccination practical ability, and payment ability, were grouped under the dimension of competence literacy in China-VLS; And subjective and affective factors, such as disease prevention self-satisfaction, trust, compliance, and collective responsibility, were categorized under the dimension of decision-making literacy (46).

The present study revealed that participants’ acceptance of the influenza vaccine in past and future intention to be vaccinated was positively associated with their total vaccine literacy levels and two of the three sub-dimensions: competence literacy and decision-making literacy. However, knowledge literacy suggested a negative or no relationship with acceptance or intention about influenza vaccination. Previous studies focused on the relationship between health literacy, vaccine literacy or educational level and vaccine acceptance, and it was indicated that the link between them and vaccination acceptance was uncertain (5, 65, 66). However, there have been few studies focusing specifically on the relationship between vaccine knowledge and vaccine uptake. It was found that health knowledge level might not be related with health outcomes. And knowledge about vaccinations did not enhance people’s engagement in deciding whether to vaccinate themselves or their children (31, 67). This is also where the limitations of applying the KAP (knowledge, attitude, and practice) theory to address public health issue (68, 69). People with a lower knowledge level were more willing to receive the vaccine because they might have higher conformity to medical advice and be more influenced by others in their decision-making process (70). And people with a higher knowledge level could help develop their perceptions regarding disease susceptibility, vaccine effectiveness and vaccination risk (55). However, using the knowledge for high involving and calculating of the pros and cons regarding vaccination can also cause an abundance of contradictory information and fence-sitting which describes a state of indecision or reluctance to make a decision, resulting in no clear preference for or against vaccination (71, 72).

It may be suggested that participants’ ability to utilize information and vaccine services, as assessed by competence literacy, along with their subjective and affective performance, as assessed by decision-making literacy, had a more positive influence on influenza vaccine acceptance than knowledge literacy. Additionally, higher levels of competence literacy and decision-making literacy could mitigate the negative influence of knowledge literacy on vaccine hesitancy. Previous studies also indicated that the public’s vaccination intention was more closely associated with their subjective norms and many social determinants (31, 67, 73). The vaccine literacy concept mirrored the idea of health literacy. It’s not just about possessing knowledge regarding vaccines, but also about establishing a simplified system for communicating and providing vaccines as an essential component of an effective healthcare system (4). Recognizing that merely acquiring knowledge does not necessarily promote vaccination, the current approach to promoting vaccination behavior and addressing vaccine hesitancy is shifting from a focus on knowledge and attitudes to the application of behavioral science (74, 75). And improving “competence literacy” and “decision-making literacy” is precisely where behavioral science can play a role. However, the authors argued that enhancing people’s scientific knowledge about vaccines is crucial. Limited knowledge about vaccines might contribute to the spread of health misinformation and was associated with lower vaccination rates (76).

As a validated vaccine literacy scale, the reliability of predicting the relationship between vaccine literacy and influenza vaccine hesitancy requires further research and verification. Lorini’s (65) systematic review identified all possible relationships, including positive association, negative association, or no association, between health literacy and vaccination, from nine reviewed papers. Hence, future multi-center, large-sample, and longitudinal studies should be conducted to enhance our understanding of the role of vaccine literacy in predicting influenza vaccine acceptance and hesitancy.

A systematic review examining barriers to intention and behavior in influenza vaccination revealed that socio-demographic variables, including gender and age among others, were frequently identified as the primary predictors. However, they were also the most inconsistent in predicting influenza vaccination (14). It was found in current study that certain socio-demographic variables, including gender, age, education, work, and family income, had significant correlations with participants’ influenza vaccination history. However, these socio-demographic variables showed no significant association with participants’ willingness to be vaccinated against influenza in the future. Additionally, stronger perceptions of influenza susceptibility and influenza vaccine effectiveness were positively correlated with both their influenza vaccination history and influenza vaccination willingness in the future. The findings may suggest that vaccine health education should be enhanced to improve the public’s vaccine health beliefs. In addition, more attention should be paid to females, individuals with a bachelor’s degree or higher, students, and those with low family income, as they were more likely to exhibit influenza vaccine hesitancy in their actual vaccination behavior.

This study had several limitations. Firstly, the cross-sectional data made it difficult to identify cause-effect relationships. Secondly, using online sample pool may introduce a selection bias in participating in the study and make it difficult to extend the research conclusions. Most of online surveys were related to low participation of people with lower educational levels and the elderly. The present study sample had the similar issue. Thirdly, the data presented in the present study was self-reported and partly reliant on the participants’ honesty and accurate memory.

## 5 Conclusion

The present study applied a validated vaccine literacy scale to assess participants’ vaccine literacy and analyzed its relationship with influenza vaccine hesitancy. The participants demonstrated sub-optimal vaccine literacy. Influenza vaccine acceptance among them was positively associated with the total vaccine literacy and two of the three vaccine sub-dimension literacy levels: “competence literacy” and “decision-making literacy”, and negatively associated with “knowledge literacy”. It may suggest that improving the public’s competence literacy and decision-making literacy could mitigate the negative impact of vaccine knowledge on vaccine acceptance, especially since some highly educated individuals may hesitate to receive vaccines after weighing the pros and cons using their acquired vaccine knowledge. Given several research limitations, future multi-center, large-sample, and longitudinal studies should be conducted to enhance the understanding of the role of vaccine literacy in predicting influenza vaccine acceptance and hesitancy.

## Data availability statement

The raw data supporting the conclusions of this article will be made available by the authors, without undue reservation.

## Ethics statement

This study was ethically reviewed and approved by the Biomedical Ethics Committee at Anhui Medical University (IRB number: 20210614). Informed consent to utilize the collected information for research purposes was obtained from all participants.

## Author contributions

LW: Conceptualization, Data curation, Formal analysis, Funding acquisition, Investigation, Methodology, Project administration, Resources, Software, Writing – original draft, Writing – review & editing. MG: Conceptualization, Data curation, Formal analysis, Investigation, Methodology, Software, Writing – original draft, Writing – review & editing. YW: Data curation, Investigation, Methodology, Software, Writing – original draft. RC: Funding acquisition, Project administration, Resources, Supervision, Writing – review & editing. XW: Funding acquisition, Project administration, Resources, Supervision, Writing – review & editing.

## Footnotes

1 It seemed a discrepancy that almost 90% of the participants have a bachelor’s degree, given that 64% of the subjects are aged 18-24. A possible explanation about this discrepancy is that, in the Chinese cultural context, some participants believe that their educational level is the degree they are studying and preparing to obtain. It is similar that a doctoral candidate in western culture is always unofficially called “doctor” in China. As we do not know the exact percentage of participants who had this kind of misunderstanding, we do not modify the participants education level in the manuscript.
